# CO_2_ sorption and regeneration properties of fly ash zeolites synthesized with the use of differentiated methods

**DOI:** 10.1038/s41598-020-58591-6

**Published:** 2020-02-04

**Authors:** Natalia Czuma, Ignasi Casanova, Paweł Baran, Jakub Szczurowski, Katarzyna Zarębska

**Affiliations:** 1 AGH University of Science and Technology, Faculty of Energy and Fuels, al. A. Mickiewicza 30, 30-059 Kraków, Poland; 2 Universitat Politècnica de Catalunya, Institute of Energy Technologies, Campus Sud, ETSEIB, Av. Diagonal, 647, 08028 Barcelona, Spain

**Keywords:** Environmental sciences, Chemistry, Materials science, Climate change

## Abstract

Production of fly ash zeolites may be an attractive method for the utilization of solid wastes from the energy sector. Different methods of synthesis often yield a variety of zeolite types, thereby affecting the properties of the resulting materials. The attention paid to carbon dioxide emission reduction technologies fully justifies the study of the sorption behaviours of fly ash zeolites synthesized by different methods. This work investigates the sorption properties of fly ash zeolites synthesized with different methods using CO_2_. Sorption capacity and adsorption isotherms were determined following the volumetric method and textural parameters were resolved according to the Dubinin-Astakhov (DA) method. The CO_2_ sorption capacity was in the range 0.24–4.16 mmol/g. The relationships between structure and sorption behaviour were studied for each synthesis method. Some strong similarities between commercial zeolites and fly ash zeolites were found. The mechanism for sorption was proved to be physisorption which is fully reversible under selected conditions. The observed trends were used to identify the best sorbent.

## Introduction

It is believed that increasing the amount of CO_2_ caused by anthropogenic emission (recently observed to exceed the 400 ppm) is leading to global climate change. Currently, many political and ecological actions are aimed at limiting CO_2_ emissions into the atmosphere. Difficulty avoiding emissions in many sectors fully justifies the efforts made to capture carbon dioxide. The most common method is based on the utilization of liquid amines for the removal of CO_2_ from flue gases. However, this process has some drawbacks due to amine degradation leading to low CO_2_ capture efficiency, high regeneration energy requirements and corrosive effects on separation equipment units^1^. Therefore, exploration of differentiated materials for CO_2_ capture is needed. One possibility is the use of technologies based on adsorption processes. Adsorbents need to be cost-effective (low cost raw material), have low heat capacity, fast kinetics, high CO_2_ adsorption capacity and selectivity, and thermal, chemical and mechanical stability under extensive cycling^2^. The most interesting adsorbents include activated carbons, zeolites and MOFs. MOFs are currently of great importance for CO_2_ capture as they have high microporosity, unlimited chemical adjustability and surface functionality compared to conventional porous zeolites and activated carbon. Although these adsorbents work well in CCS technologies, they may not be sufficient in post-combustion processes due to very high temperatures. Additionally, MOFs are extremely expensive relative to zeolites and activated carbon; the price is estimated to be approximately $130–200/kg^3^. Activated carbon is one of the most widely used solid adsorbents for the capture of CO_2_. This is due to its microporous character, adequate pore size distribution, low cost and easy regeneration. However, activated carbon CO_2_/N_2_ selectivity is relatively low. For the purpose of adjusting the properties of activated carbons to the carbon dioxide sorption, modifications to the surface may be needed, increasing the cost of those materials. Zeolites have been proposed as potential physical adsorbents of carbon dioxide and their adsorption efficiencies depend on size, charge density, and chemical composition of cations in their porous structures^4^. Carbon dioxide capture using zeolites has certain advantages over capture processes that use carbon-based sorbents. One advantage is the higher selectivity of carbon dioxide over nitrogen, which leads to the production of highly purified carbon dioxide streams. The price of commercial zeolite starts at $100/kg. Natural zeolites are the cheapest of all the above mentioned materials, however, it should be noted that availability of this natural resource is limited. An alternative method for the production of zeolites is to use fly ash as the raw material since fly ash chemical composition is often appropriate for this purpose^5^. Fly ash zeolites are inexpensive adsorbents and their production process enables complete utilization of the waste^6^. Since the early research on zeolite synthesis from fly ash^7^, a large number of studies have been carried out to explore different synthesis methods, including classic alkaline conversion, alkaline fusion, dry (molten salt) conversion and a two-step process (e.g., see Querol *et al*., 2002, and references therein). Each of the proposed methods has advantages and disadvantages. For instance, zeolite production by the fusion method is efficient, but the process is complicated and energy-demanding; the dry conversion allows a reduction of waste generated in the process, but synthesized zeolites display properties that significantly limit their utilization. The cost of hydrothermally synthesized zeolites on half-industrial scale was estimated to be between $40–96/kg^8,9^. In the case of two-step synthesis, the cost would be similar. There are no data concerning the cost of fly ash zeolite synthesis using the fusion method on a larger than laboratory scale. As the technology for selection and modification of adsorbents advances, new methods of synthesis will be developed.

In this paper, zeolites were synthesized with the use of hydrothermal (H), fusion (F) and a modified two-step (TS) process. The sorption properties and production yield were evaluated for each method.

## Methods

Zeolites were synthesized from fly ash raw materials following three different methods: hydrothermal (Zarębska *et al*., 2015), fusion (Czuma *et al*., 2017) and a modified two-step procedure (Czuma *et al*., 2019).

Major and minor element contents were determined by X-ray fluorescence (XRF) analysis with a Philips PW 1404 spectrometer. Mineral identification was carried out by powder X-ray diffraction (XRD) using a Philips X’pert APD diffractometer (with a PW 3020 goniometer), with Cu cathode and a graphite monochromator (in a 2θ degree range of 5–50°, with a step-size of 0.02° and measuring time of 1 s per step). A Thermo Scientific Nicolet 6700 FT-IR spectrometer equipped with a DTGS-CsI detector and operating across a spectra range from 400 cm^−1^ to 4000 cm^−1^, 64 scans and 4 data point spacing was used for infrared spectroscopy characterization. Scanning electron microscopy was performed with a Neon 40 Crossbeam workstation, operating at 15 keV.

Specific surface area (S_BET_) measurements of raw fly ash and synthesized zeolites were performed with a Micrometrics ASAP 2020 analyser and calculated using the linear form of the Brunauer-Emmett-Teller (BET) equation, assuming an area of 0.162 nm^2^ for the N_2_ adsorbed molecule. Nitrogen adsorption-desorption isotherms were determined at liquid nitrogen temperature (77 K). Sorption experiments using the volumetric method were carried out using equipment for precise physical sorption and chemisorption measurements, with a vapour adapter and an Autosorb-1-C mass spectrometer (Quantachrome Instruments, USA). Samples were pre-heated to 473 K for 12 hours using a degasser equipped with a vacuum system with a turbomolecular pump. Measurements were carried out at room temperature. Thermogravimetric analyses (TGA) were performed with a TA Instruments Q50 analyser. Samples of about 10 mg were placed in the furnace, heated to 458 K (10 K/min) in an inert nitrogen atmosphere, kept at constant temperature for 20 minutes and then cooled down to 323 K. When the temperature reached 323 K the gas flow was switched to carbon dioxide and kept for 40 minutes under isothermal conditions. This procedure was repeated for three sorption-desorption cycles.

## Results

### Materials characterization

Fly ash resulting from the combustion of a pulverized hard coal were collected at the cumulative container of the Polish power plant. These materials can be classified as a standard F class fly ash, according to ASTM C618 (2019). The elemental composition determined by XRF is shown in Table 1.

**Table 1:** Oxide composition of raw fly ash from the Polish power plant used for zeolite synthesis.

Component	Concentration, %
SiO_2_	38.9
Al_2_O_3_	20.3
Fe_2_O_3_	9.0
CaO	7.7
MgO	3.3
Na_2_O	3.0
K_2_O	2.9
SO_3_	1.8
TiO_2_	1.2

**Table 2:** Comparison of S_BET_ values for examined samples.

Sample symbol	S_BET_, m^2^/g	Micropore volume (D-R), cm^3^/g	Total pore volume at p/p_0_ = 0.95, cm^3^/g	% of micropore volume
fly ash	2	—	—	—
H	39	0.016	0.138	12
F	414	0.188	0.251	75
TS	34	0.013	0.069	19

**Figure 1:**
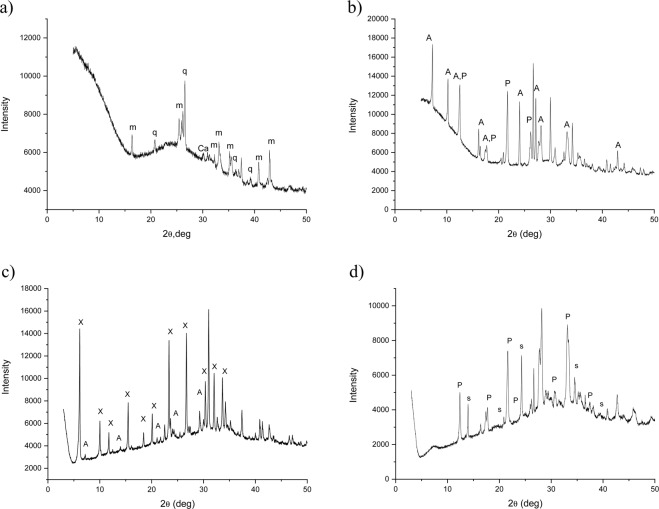
XRD analyses of raw fly ash (**a**) and samples synthesized by the H (**b**), F (**c**) and TS (**d**) methods (labels: m = mullite; q = quartz; Ca = calcite; X = zeolite; A = zeolite A; P = zeolite P1; s = sodalite).

The mineralogical (XRD) study of the raw materials shows that the fly ash is mainly composed of mullite and quartz, with some calcite (Fig. 1a). The increase of the intensity of the XRD halo pattern in the 2θ range between 20° and 35° suggests the presence of a significant amorphous component in the sample^10^. Treatment with different synthesis methods yielded the formation of at least three different types of zeolites: A and P1 in the hydrothermal method, A and X with the fusion method, P1 and sodalite when fly ash samples were subjected to the two-step zeolite synthesis method (Fig. 1b–d, respectively).

**Figure 2:**
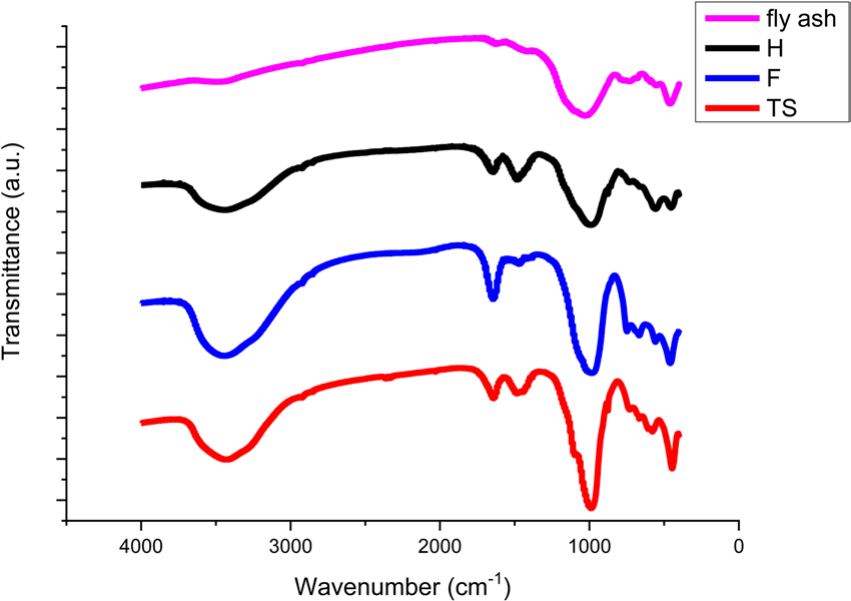
IR analysis of starting fly ash and synthesized zeolites.

Characterization of the materials by FTIR (Fig. 2) shows the differences between the starting fly ash and the materials synthesized, where the following zeolite characteristic bands can be observed: bands at around 3500 cm^−1^ are associated with the stretching vibration of the hydroxyl groups –OH; bands at 1600 cm^−1^ are characteristic of the bending mode in the water molecule; bands at 1000 cm^−1^ are related to the asymmetric internal T–O (T = Si, Al) stretching vibrations of the TO_4_ primary building units; bands at 561 cm^−1^ can be attributed to the D6R T–O–T symmetric stretching; a sharp band at 460 cm^−1^ corresponds to the Si–Al–O bending mode^11–15^.

**Figure 3:**
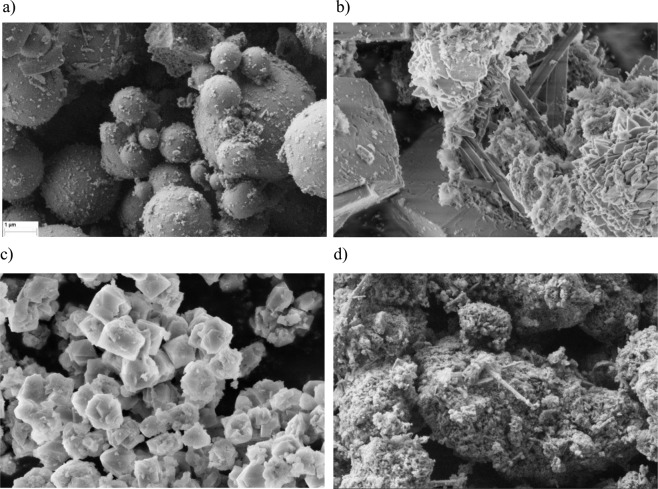
Representative SEM images of raw fly ash (**a**), and zeolites synthesized by the hydrothermal (**b**), fusion (**c**) and two-step (**d**) methods.

SEM imaging of the fly ash (Fig. 3a) reveals the predominance of aluminosilicate glass spherical particles. All post-synthesis samples display significant changes in their microstructures: cubic crystals characteristic of zeolite A (Fig. 3b), needle-shaped zeolite P1 (Fig. 3b,d) and complex zeolite X octahedra (Fig. 3c), as identified by Fotovat *et al*., (2009), Koshy and Singh (2016) and Shigemoto and Hayashi (1993), respectively. It is evident that not many unreacted fly ash particles remain after any of the synthesis processes and that the surface of post-synthesis materials is much rougher than the raw material.

**Figure 4:**
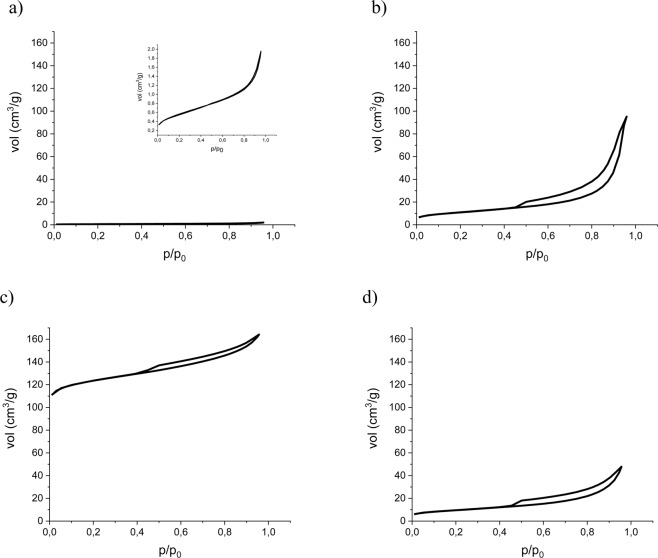
Low-temperature nitrogen adsorption isotherm of raw fly ash (**a**), and zeolites synthesized by the hydrothermal (**b**), fusion (**c**) and two-step (**d**) methods.

Low temperature nitrogen adsorption isotherms are presented in Fig. 4.

The following textural parameters were calculated from low-temperature (77 K) nitrogen adsorption isotherms: specific surface area (BET model), micropore volumes (Dubinin Radushkevich model-DR) and total pore volume at p/p_0_ = 0.95. The results are shown in Table 2.

The specific surface area parameter indicates that all synthesis methods yield zeolites with higher values of S_BET_ compared to the raw fly ash (by about a factor of 20 in the H and TS methods and 200 times more than the original surface area after the F synthesis). Micropore pore volumes (MPV) follow a similar trend, with the value for sample F being about 10 times higher than for H and TS. The MPV/S_BET_ ratios are fairly constant, (3.2–4.1 × 10^−4^), suggesting that dissolution (melting) and precipitation of single crystals (as opposed to, for example, diffusional growth) is most likely to account for the conversion of fly ash into zeolite in all cases.

The adsorbents should have an optimum pore size distribution for the best possible access of the gas molecules through the nano-pores. The most common adsorbents are composed of a combined network of micro, meso and macropores. The only exception is zeolites; their pores are precisely defined.

The affinity between adsorbate and adsorbent is estimated based on the slope of isotherms at low pressures. The best material should have a steep isothermal slope which indicates high CO_2_ sorption capacity at low concentrations. A milder slope angle of the carbon dioxide adsorption isotherms is associated with lower capture.

### CO_2_ sorption experiments

Carbon dioxide sorption experiments were carried using two different methods providing complementary information. The aim was to carry out a comparative study of sorption capacity, textural parameters and preliminary regeneration possibilities between synthesized materials and two commercial zeolites. Such a comparison enabled the evaluation of their potential utilization.

The pore size distribution (PSD) of samples was determined through measurement of a CO_2_ adsorption isotherm. PSD measurements are commonly performed using nitrogen or argon at 77 K and 87 K, respectively. At these low temperatures, kinetic limitations can have an effect on the process reaching equilibrium during isotherm measurements.

The study of the adsorption of a wide range of microporous materials is commonly addressed following the equation proposed by Dubinin and Astakhov (1971) (Eq. 1):

1 $$W={W}_{0}exp\,\left[-{\left(\frac{-RTlnP/{P}_{0}}{E}\right)}^{n}\right]$$

where:

*W* – weight adsorbed at P/P_0_ and T

*W*_0_ – total weight adsorbed

*E* – characteristic energy of adsorption (*E* = *E*_0_ *β*)

*E*_0_ – characteristic energy of adsorption for a standard vapour

*β* – coefficient of affinity

*n* – non-integer value (typically between 1 and 3)

This approach requires the calculation of *n* and *E* parameters by non-linear curve fitting.

The values can then be used to determine the pore size distribution of the material through the application of the expression proposed by Medek^16^:

2 $$\frac{d\left(\frac{W}{{W}_{0}}\right)}{dr}=3n{\left(\frac{k}{E}\right)}^{n}{r}^{-(3n+1)}exp\,\left[-{\left(\frac{k}{E}\right)}^{n}{r}^{-3n}\right]$$

where *k* is the interaction constant for CO_2_ and N_2_, the values are typically 1.5 and 2.96 kJ·nm^−3^·mol^−1^, respectively, with a pore radius r.

**Figure 5:**
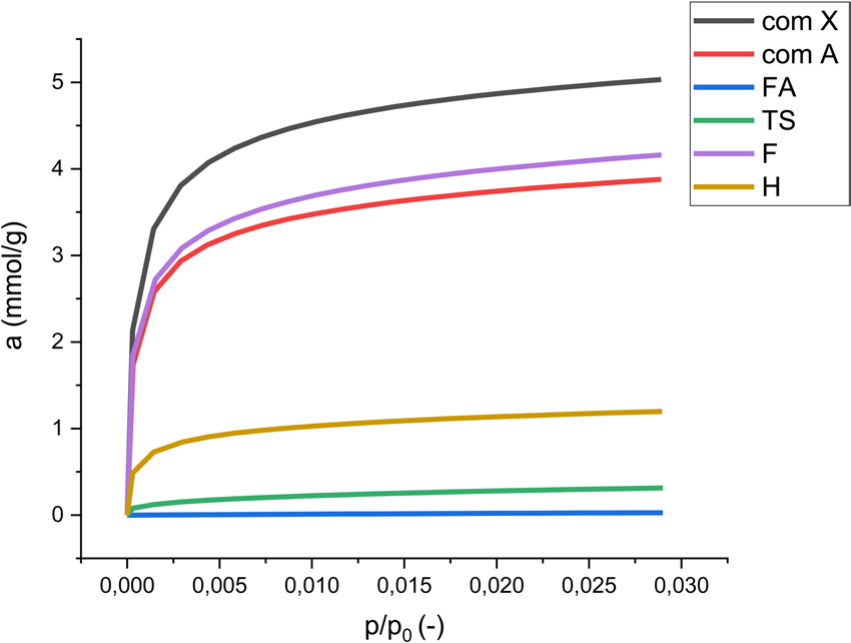
Volumetric sorption experiment performed on unprocessed fly ash (FA), H fly ash zeolite sample (H), F fly ash zeolite sample (F), TS fly ash zeolite sample (TS), commercial zeolite A (com A), commercial zeolite X (com X).

The results of volumetric tests are presented on the adsorption isotherm in Fig. 5, where a is the adsorption capacity value and p/p_0_ is relative pressure.

**Figure 6:**
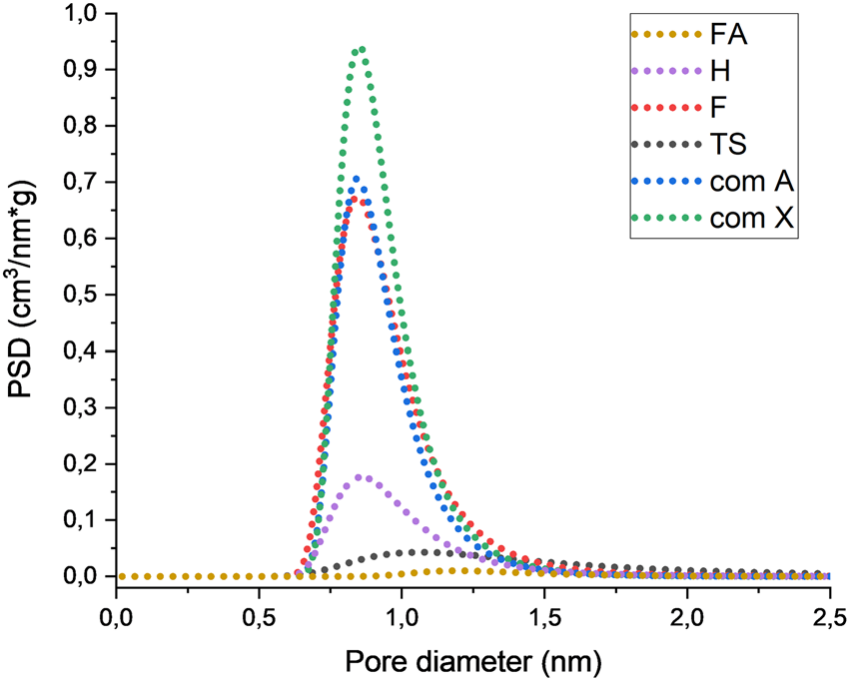
Differential pore size distribution (PSD) calculated form DA for unprocessed fly ash (FA), H fly ash zeolite sample (H), F fly ash zeolite sample (F), TS fly ash zeolite sample (TS), commercial zeolite A (com A), commercial zeolite X (com X).

Carbon dioxide isotherms were used to calculate sorption capacities (Table 3) and the differential pore distribution dV/dD was calculated using the Dubinin-Astakhov method (Fig. 6).

**Table 3:** Comparison of results from volumetric sorption of carbon dioxide test with textural parameters calculated from carbon dioxide isotherms.

Sample symbol	CO_2_ sorption capacity, mmol/g	DA Micropore volume, cm^3^/g	S_DA_ Surface area, m^2^/g	DA Pore diameter, nm
fly ash	0.03	0.006	11	1.2
H	1.20	0.070	162	0.86
F	4.16	0.227	549	0.84
TS	0.24	0.043	50	1.06
Commercial zeolite A	3.88	0.202	506	0.84
Commercial zeolite X	5.03	0.261	652	0.84

**Figure 7:**
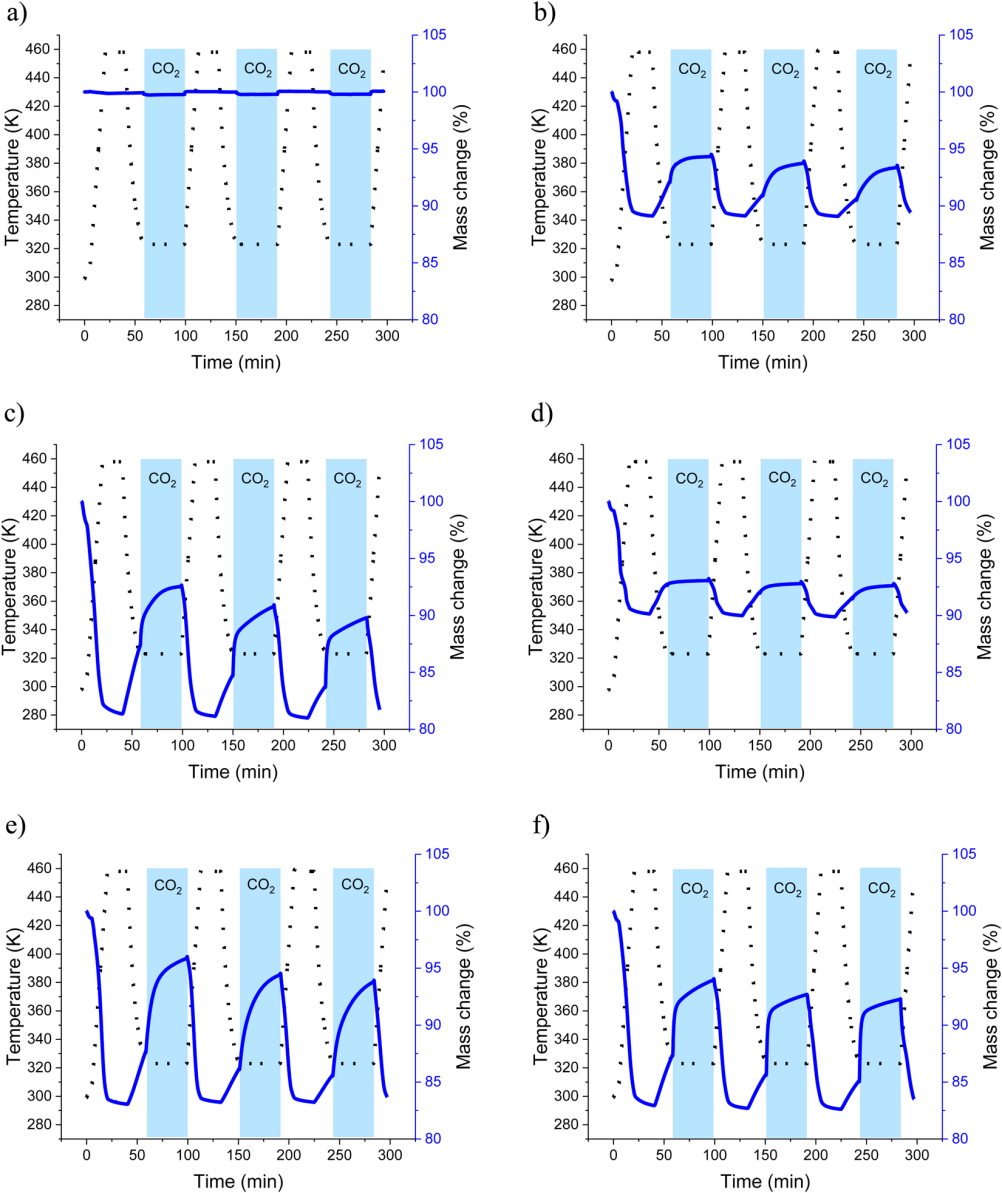
Temperature (dotted) and mass change (solid) evolution of sorption experiments performed on unprocessed fly ash (**a**), H zeolite (**b**), F zeolite (**c**), TS zeolite (**d**), commercial zeolite A (**e**), and commercial zeolite X (**f**).

Characterization of sorption properties was carried out by TGA, the results are presented in Fig. 7. A sorption temperature of 323 K was chosen based on previous work on zeolite 4 A^17^. Desorption temperature (458 K) was chosen on the assumption that such a temperature can easily be attained in industrial applications using waste heat.

## Discussion

Zeolites synthesized from fly ash by different methods showed different conversion efficiencies and carbon dioxide sorption capacities. Volumetric measurements indicated that the fusion method (F) yielded the best sorption capacity, approximately three times higher than the hydrothermal (H) method and almost 20 times higher than the two-step synthesis (TS). The sorption capacity of both F and H samples increases rapidly at low p/p_0_ values, indicating the microporous character of the sample surface. This is confirmed by the high surface area calculated from both N_2_ and CO_2_ measurements, accounting for the observed high conversion ratio of fly ash into crystalline zeolite. The isotherms of samples H and F are similar to those of commercial zeolites A and X. Unprocessed fly ash does not show any evidence of carbon dioxide sorption properties.

The values of specific surface area calculated from low temperature nitrogen sorption S_BET_ and the values calculated from the carbon dioxide sorption S_BETCO2_ cannot be compared. However, common trends can be observed. In the case of S_BET,_ the values obtained for samples H and TS are of the same order. Once the analogical comparison is made for S_BETCO2,_ the value obtained for H is four times higher. Such observation proves the microporous character of sample H. It can be stated that in the case of microporous materials, the S_BET_ analysis with the use of low temperature nitrogen sorption may not give sufficient data for comparison.

Differential pore size distribution (PSD) calculated from the DA method presented in Fig. 6 represents the trend of decreasing sorption properties along with the decreasing number of micropores. This proves that the presence of the micropores determines the effectiveness of the sorbent. DA pore mean diameter values presented in Table 3 show that in the case of commercial zeolites and samples H and F, the mean diameter is relatively low. In the case of unprocessed fly ash, the value of 1.2 nm should be interpreted as intergrain spaces, since fly ash, (as will be proven in the following section), does not exhibit porous characteristics. The same approach could be applied to sample TS.

Mass change evolution in sorption experiments monitored by TGA shows an initial drop for all zeolites analysed, mainly due to desorption of water molecules present in intercrystalline voids. It may be assumed that a higher mass decrease in the initial stages of the experiment indicates a higher content in the zeolite phase. Sample F, together with commercial zeolites, shows the larger desorption (more than 15% of the original mass), followed by samples H and TS (both at about 10% mass loss), indicating the higher conversion rate of fly ash into zeolite by the fusion method.

TGA of samples H and F showed a relatively high increase in mass after the gas inlet was switched to CO_2_. No such an increase was observed in sample TS. This may be due to the low conversion rate of fly ash into zeolite of the two-step method, and/or the presence of different types of zeolites in the samples. As evidenced by XRD analysis, sample TS contains type P1 zeolites and sodalite. Due to the presence of very small pores, neither of these substances is believed to have a good sorption capacity^18,19^.

On the other hand, when CO_2_ is introduced in the system, commercial zeolite X experiences a lower mass gain compared to commercial zeolite A despite the fact that its micropores are larger than those in zeolite A. This behaviour has also been reported in previous work (e.g., *Bukalak et al*., 2012).

It should be noted that both the increasing amount of micropore share in the pore volume and the presence of different metal phases of fly ash zeolites, provides stronger CO_2_/N_2_ selectivity (Zgureva, 2016). It is confirmed in the literature that commercial zeolites A and X present CO_2_ selectivity over N_2_ (Myers, 1973).

There are some indications that the CO_2_ affinity of zeolites may also be connected with their Si/Al ratio^20^; the lower value of this parameter the better carbon dioxide sorption properties^21^.

In TGA experiments with samples F and H, very small decreases in mass sorbed (~2%) were observed at each cycle, suggesting good regeneration possibilities. Although the mass seemed to remain essentially unchanged after regeneration, the slight decrease may be associated either with remains of physisorbed CO_2_ (which may be responsible for blocking entrances to the pore network), or to slight structural changes taking place during the heating process. It must be taken into account that chemisorption may also occur, resulting in the formation of carbonates and carboxylates^21^. This has been attributed to the presence of unreacted calcium oxide, however, no such phases have been identified in this work.

Analogous experiments were performed using a heating temperature of 573 K (see Supplementary Appendix 1). The outcome of these experiments proved that the use of a higher temperature avoids decreases in sorption capacity, as the mass of samples in the following cycles did not change. This is complementary information indicating that the use of higher temperatures allows for full regeneration and maximum cycling capacity, also confirmed that the process of binding of CO_2_ is physisorption.

The results from the experimental procedure presented in this work indicate that unprocessed fly ash does not have any significant sorption capacity. Samples H, F, commercial zeolite A and commercial zeolite X, seem to have relatively short CO_2_ saturation times^22^.

The SEM study revealed clear textural differences among the zeolites synthesized by different methods. Although this is difficult to correlate quantitatively with the physical parameters determined by the other experimental and characterization procedures, it is evidence that the microstructural basis is the explanation for the observation that the fusion method yields the highest conversion efficiency of all the methods studied in this work.

## Conclusions

Compared to unprocessed fly ash, fly ash zeolites exhibit sorption capacity related to carbon dioxide. The sorption capacity was the lowest for the TS sample (0.24 mmol/g), the H sample reached a value of 1.2 mmol/g, and the highest for fly ash zeolite was observed in the F sample with a value of 4.16 mmol/g. The changes in the value occur according to the synthesis method and can be associated with textural parameters like the S_BET_ value (sample TS 34 m^2^/g, sample H 39 m^2^/g, sample F 414 m^2^/g). Similarities of fusion synthesized materials and commercial zeolites can be observed, proving that this synthesis method is highly efficient. Specific surface area calculations with the use of two gases provide information on the microporous character of the sample, indicating that nitrogen low temperature sorption may be insufficient to compare samples. Pore size distribution proves that the presence of the micropores determines the efficiency of the sorbent. Both volumetric and thermogravimetric tests indicate that a fusion synthesized sample, followed by hydrothermally synthesized sample present very good and good sorption capacity characteristics, two step synthesized sample sorption properties are much lower. The reason for this is the presence of different types of zeolites in the samples, the Si/Al ratio and the yield of zeolite present in the sample. Thermogravimetric tests provide information about regeneration properties of analysed samples, regarding the energy penalty for the purpose of regeneration. This information also confirms that a physical adsorption process is taking place. Comparing structure with the sorption results provides evidence of a correlation between the amount of crystals and sorption capacity.

## Supplementary information

Supplementary Information.
