# Supplementary material for: CO2 sorption and regeneration properties of fly ash zeolites synthesized with the use of differentiated methods
Source: Sci Rep. 2020 Feb 4;10:1825. doi: 10.1038/s41598-020-58591-6 (PMC7000786; doi:10.1038/s41598-020-58591-6)
Supplement: Supplementary file 1 — Supplementary Information. [file 41598_2020_58591_MOESM1_ESM.docx]

Supplementary appendix 1: Temperature (dotted) and mass change (solid) evolution of sorption experiments performed on a F zeolite sample, heated up to temperature 573 K
